# Prepackaged formula low-residue diet vs. self-prepared low-residue diet before colonoscopy: A multicenter randomized controlled trial

**DOI:** 10.3389/fmed.2023.1029493

**Published:** 2023-03-24

**Authors:** Peng Pan, Lun Gu, Shengbing Zhao, Shuling Wang, Jiayi Ma, Hongyu Fu, Youxiang Chen, Shuixiang He, Zibin Tian, Le Xu, Zhijie Feng, Yanqing Li, Zhuo Yang, Lei Yang, Wen Wang, Qian Hou, Ting Liu, Chujun Li, Dean Tian, Xiaodi Wang, Yongmei Gao, Hanping Shi, Yu Bai, Zhaoshen Li

**Affiliations:** ^1^Department of Gastroenterology, Changhai Hospital, Second Military Medical University, Naval Military Medical University, Shanghai, China; ^2^Department of Gastroenterology, The First Affiliated Hospital, Nanchang University, Nanchang, China; ^3^Department of Gastroenterology, The First Affiliated Hospital, Xi'an Jiaotong University, Xi'an, China; ^4^Department of Gastroenterology, The Affiliated Hospital, Qingdao University, Qingdao, China; ^5^Department of Gastroenterology, Beijing Hospital, Beijing, China; ^6^Department of Gastroenterology, The Second Hospital, Hebei Medical University, Shijiazhuang, China; ^7^Department of Gastroenterology, Qilu Hospital, Shandong University, Jinan, China; ^8^Digestive Endoscopy Center, Northern Theater General Hospital, Shenyang, China; ^9^Department of Gastroenterology, The 900th Hospital of the Chinese People's Liberation Army (PLA), Fuzhou, China; ^10^Department of Nutrition, Xiangya Hospital, Central South University, Changsha, China; ^11^Department of Gastroenterology, Xiangya Hospital, Central South University, Changsha, China; ^12^Department of Gastrointestinal Endoscopy, The Sixth Affiliated Hospital, Sun Yat-sen University, Guangzhou, China; ^13^Department of Gastroenterology, Tongji Hospital, Tongji Medical College of Huazhong University of Science and Technology, Wuhan, China; ^14^Department of Gastroenterology, China-Japan Friendship Hospital, Beijing, China; ^15^Department of Gastroenterology, The First Affiliated Hospital, Hebei North University, Zhangjiakou, China; ^16^Department of Gastrointestinal Surgery, Beijing Shijitan Hospital, Capital Medical University, Beijing, China

**Keywords:** low-residue diet, bowel preparation, dietary restriction, colonoscopy, adenoma detection rate

## Abstract

**Background and aims:**

Compared with self-prepared LRD, a prepackaged low-residue diet (LRD) can improve patient compliance, but whether it can further improve the quality of bowel preparation is uncertain. The study aimed to compare the application of the prepackaged formula LRD with self-prepared LRD in bowel preparation for colonoscopy.

**Methods:**

A multicenter randomized controlled trial was conducted in 15 centers. The eligible subjects were randomly assigned to one of two groups: the formula LRD group and the self-prepared LRD group. On the day before the colonoscopy, subjects in the self-prepared LRD group were instructed to consume a restricted LRD prepared by themselves, while subjects in the formula LRD group were given six bags of prepackaged formula LRD and instructed to consume them according to their individual need. The primary outcome was an adequate bowel preparation rate. Secondary outcomes mainly included Boston Bowel Preparation Scale (BBPS) scores, dietary restriction compliance rate, tolerance, satisfaction, adenoma detection rate (ADR), and adverse reactions. The trial was registered at ClinicalTrials.gov under the identifier NCT03943758.

**Results:**

A total of 550 subjects were recruited. Compared with the self-prepared LRD group, the formula LRD group showed a higher adequate bowel preparation rate (94.5 vs. 80.4%; *P* < 0.01), BBPS scores (7.87 ± 1.13 vs. 6.75 ± 1.47; *P* < 0.01), dietary compliance rate (92.4 vs. 78.9%; *P* < 0.01), tolerance (*P* < 0.01 in degree of hunger, intensity of physical strength, and negative influence on daily activities), satisfaction (8.56 ± 1.61 vs. 7.20 ± 2.02; *P* < 0.01), and ADR (25.6 vs. 16.0%; *P* < 0.01). There was no significant difference in adverse reactions.

**Conclusion:**

Compared with self-prepared LRD, the formula LRD showed similar safety and higher bowel preparation quality, compliance, and tolerance in bowel preparation. More formula LRDs could be designed according to different dietary habits and ethnic populations, and further researches are warranted to confirm their effect.

**Clinical trial registration:**

https://register.clinicaltrials.gov, identifier: NCT03943758.

## Introduction

Colonoscopy is an important diagnostic modality for colorectal diseases and the gold standard of colorectal cancer (CRC) screening. The diagnostic accuracy of colonoscopy largely depends on the quality of bowel preparation (1, 2). During a colonoscopy, adequate bowel preparation facilitates clear mucosal visualization, while inadequate bowel preparation may result in negative impacts, including lower adenoma detection rate (ADR), lower cecal intubation rate, longer procedural time, shorter surveillance interval, and higher risk of canceled procedure (3–5). However, previous studies have reported inadequate bowel preparation rates as high as 18–35%, with adequate bowel preparation rates well below the minimum standard of 90% (6, 7).

Dietary restriction helps reduce the production of fecal residue and is one of the important steps in the bowel preparation process (8, 9). Guidelines recommend a low-residue diet (LRD) on the day preceding colonoscopy (1, 2). To improve patient compliance with LRD, prepackaged LRD was introduced, and prior studies have shown that, compared with self-prepared LRD, prepackaged LRD can significantly improve patient compliance, but whether it can further improve the quality of bowel preparation is uncertain (10–14). Prepackaged LRDs used in previous studies were composed of unrefined conventional foods, which may inevitably contain dietary fiber or take on shapes or structures of the foods themselves, thus potentially affecting the bowel preparation quality to some extent (10–13, 15).

To further improve the quality of bowel preparation, a prepackaged formula LRD was specifically designed, which was the first Food for Special Medical Purpose (FSMP) approved for bowel preparation for colonoscopy in China. Dietary fiber was completely eliminated from the formula LRD through nutritional technology and the formula LRD was processed to powder form for better digestion and absorption. In addition, the formula LRD contained sufficient energy, a balanced proportion of carbohydrates, fats, and proteins, and rich vitamins and micronutrients. The recommended daily intake of six bags of the formula LRD provided 6,540 kilojoules of energy, which accounts for 68% of recommended daily intake for young and middle-aged men with light physical activity in China. The purpose of the study was to comprehensively compare the application of the prepackaged formula LRD with self-prepared LRD in bowel preparation based on polyethylene glycol (PEG) for colonoscopy in a Chinese adult population.

## Methods

### Overall design

The colonoscopists-blinded, multicenter, randomized controlled trial was invited by the National Clinical Research Center for Digestive Diseases (Shanghai) and the National Quality Control Center of Digestive Endoscopy in China, and was conducted in 15 medical centers: Changhai Hospital of Naval Military Medical University, the First Affiliated Hospital of Nanchang University, the First Affiliated Hospital of Xi'an Jiaotong University, the Affiliated Hospital of Qingdao University, Beijing Hospital, the Second Hospital of Hebei Medical University, Qilu Hospital of Shandong University, Northern Theater General Hospital, the 900th Hospital of the PLA, Xiangya Hospital of Central South University, the Sixth Affiliated Hospital of Sun Yat-sen University, Tongji Hospital of Tongji Medical College, China-Japan Friendship Hospital, the First Affiliated Hospital of Hebei North University, and Beijing Shijitan Hospital of Capital Medical University. The trial was designed according to the Consolidated Standards of Reporting Trials guidelines and registered online at ClinicalTrials.gov (NCT03943758). The study protocol conformed to the Declaration of Helsinki and was approved by all participating medical centers. Prior to enrolment, all potential subjects were informed about the background, objective, procedures, benefits, and likely risks associated with their participation in the trial.

### Population, randomization, and blinding

Subjects who satisfied the following inclusion criteria were potentially eligible: (i) Chinese and aged 18–65 years, (ii) scheduled for diagnostic, screening, or surveillance colonoscopy, and (iii) able to fill in the informed consent form for participation.

Subjects with heart failure, stroke or renal failure, a history of colon surgery, inflammatory bowel disease, digestion or absorption dysfunction, gastrointestinal tract obstruction, any dietary restriction due to various reasons, a history of hypersensitivity to any ingredients of laxatives or soy products, and high-risk factors may affecting bowel preparation such as constipation, body mass index more than 30 or <18 kg/m^2^, diabetes, spinal cord injury, or use of medications affecting bowel motility within a week were excluded. In addition, pregnant or lactating subjects were excluded.

At each medical center, eligible subjects were randomly assigned into one of the two dietary restriction groups based on a randomization table: the self-prepared LRD group or the formula LRD group. The randomization table was centrally generated by the Center for Clinical Epidemiology (Naval Medical University). Allocation concealment was achieved using sequentially numbered sealed opaque envelopes. The researcher who generated the randomization table and the researchers who instructed subjects to implement dietary restrictions were not involved in the colonoscopy procedure.

### Bowel preparation regimens

On the day before the colonoscopy, subjects in the self-prepared LRD group were instructed to restrict their diet to rice porridge, rice soup, noodles, and eggs. Subjects in the formula LRD group were given six bags (60 g per bag) of prepackaged formula LRD (Maifu Nutritional Technology Co., Ltd., China) and were instructed to consume them according to their individual need. If the prepackaged formula LRD was insufficient or unacceptable, subjects in the formula LRD group would be allowed to consume the food of the self-prepared LRD group. The main raw ingredients of the prepackaged formula LRD included the following: maltodextrin, crystallized fructose, soybean protein isolate, vegetable oil, medium chain triglyceride, and rice protein powder. The nutritional information of the prepackaged formula LRD is shown in Supplementary Table 1. The method to use the formula diet are as follows: pour the powder into a container, add warm water, and stir well-before eating, 1–2 bags each time. Each bag of the formula LRD required about 250 ml of warm water.

All subjects adopted the same catharsis regimen as follows: ingested 1,000 ml PEG solution (Shenzhen Wanhe Pharmaceutical Co., Ltd., Shenzhen, China; or Staidson Biopharmaceuticals Co., Ltd., Beijing, China) at 8 p.m. the day before colonoscopy at a rate of 250 ml per 10–15 min, and ingested 2,000 ml PEG solution plus 30 ml simethicone (Menarini Pharmaceutical Co., Ltd., Italy) 4–6 h prior to colonoscopy at the same rate.

### Questionnaire survey

Approximately 5–10 min prior to colonoscopy, subjects were assisted to complete a questionnaire about their experience of bowel preparation. Questionnaire items included compliance with dietary restrictions, the volume of PEG intake, related adverse reactions (nausea, vomiting, bloating, abdominal pain, insomnia, and allergy), tolerance (degree of hunger, intensity of physical strength, and influence of dietary restrictions on daily activities), and satisfaction with the overall bowel preparation process. Visual analog scales were used to assess tolerance and satisfaction with the highest score of 10 indicating great satisfaction or tolerance and the lowest score of 0 very poor. In addition, subjects in the formula LRD group were asked to evaluate the taste (poor, average, good, or excellent) of the prepackaged formula LRD.

### Colonoscopy

In each medical center, colonoscopies were performed by assigned colonoscopists (with experience of >1,000 colonoscopy procedures) blinded to the group of subjects. The colonoscopists received unified training on bowel preparation quality scoring prior to the study. The colonoscopy withdrawal time should not be <6 min, excluding time for biopsy performance or removal of polyps. Biopsies were performed when suspected lesions were observed, and the final diagnosis was based on pathological examination.

### Outcomes

The primary outcome was the rate of adequate bowel preparation. Adequate bowel preparation was defined as a score of ≥2 on all colon segments of the colon (right, transverse, and left colon) based on the Boston Bowel Preparation Scale (BBPS) (16). Secondary outcomes included total BBPS scores, the BBPS score of each segment of the colon, excellent bowel preparation (BBPS score = 9) rate, dietary restriction compliance rate, adverse reactions, PEG compliance (PEG intake ≥ 80%) rate, tolerance, satisfaction, ADR, cecal intubation time, and withdrawal time. To calculate the dietary restriction compliance rate, the number of subjects who adhered to dietary restriction was divided by the total number of subjects in the corresponding group. In the self-prepared LRD group, subjects who consumed medium and high-residue foods other than rice soup, noodles, porridge, and eggs the day before the colonoscopy were considered non-compliant. To calculate ADR, the number of colonoscopies in which at least one adenoma was detected and pathologically confirmed was divided by the total number of colonoscopies in the corresponding group.

### Sample size calculation and statistical analysis

According to the preliminary trial, the rate of adequate bowel preparation was ~80% in the self-prepared LRD group, thus, an estimated sample size of 263 patients in each arm would provide a power of 90% to detect a 10% increase in the rates of adequate bowel preparation from the self-prepared LRD group to the formula LRD group, but when a 10% dropout rate was considered, at least 289 patients were needed in each arm.

Statistical analyses were performed using IBM SPSS Statistics for Windows, Version 21.0 (Armonk, NY: IBM Corp.). Categorical variables were calculated as frequencies and percentages and compared by Pearson's chi-square test or Fisher's exact test. Continuous variables were calculated as the mean and standard deviation and compared by the two-sided Student's *t*-test. Ranked data were tested by the Wilcoxon–Mann–Whitney test. Both intention-to-treat (ITT) analysis and per protocol (PP) analysis were conducted. Subgroup analysis was performed for subjects aged 45 years or older because 45 is the recommended initiating age for CRC screening. A *p*-value <0.05 was considered statistically significant.

## Results

### Study population and baseline characteristic

**Figure 1:**
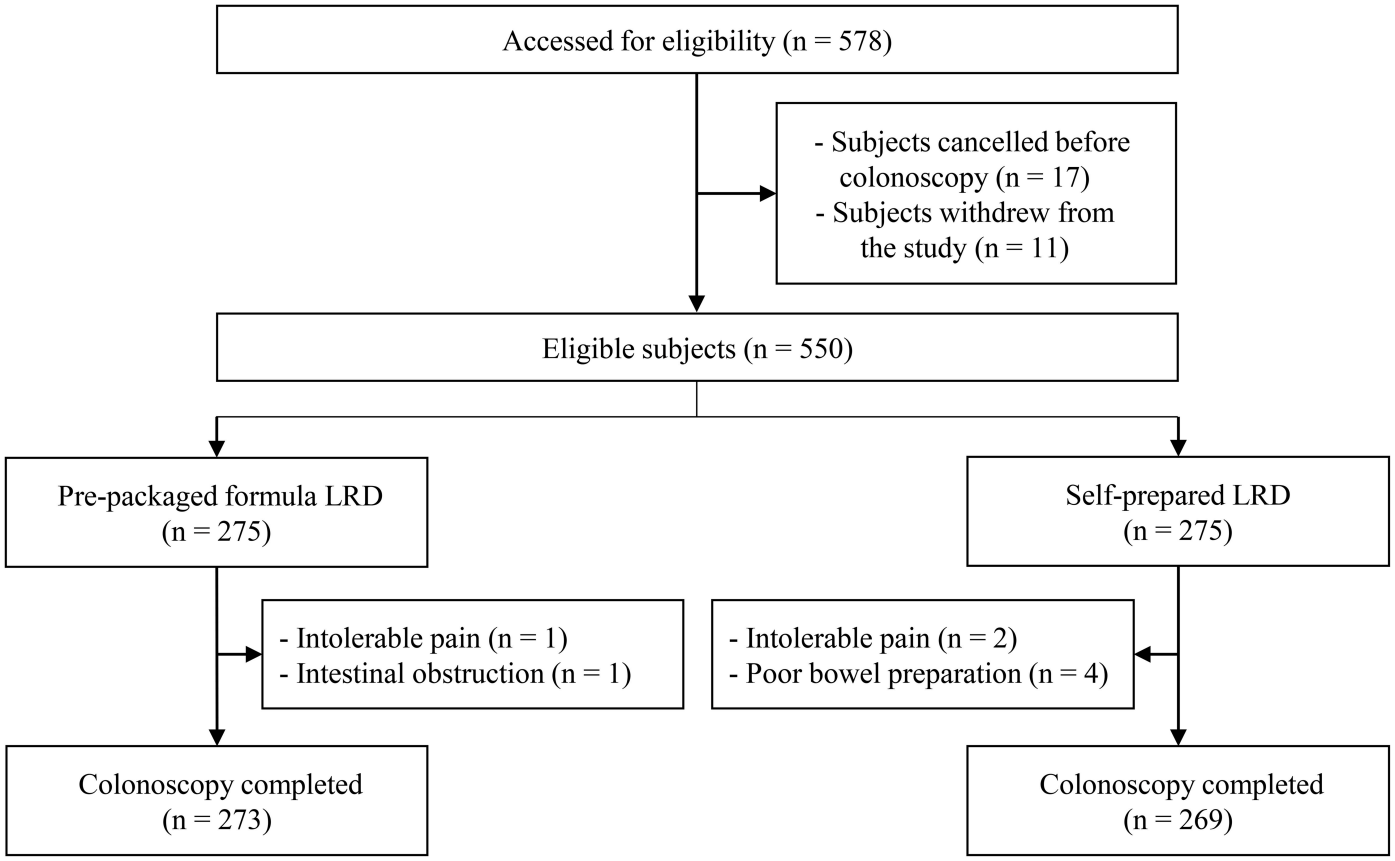
Study flow diagram. LRD, low-residue diet.

The trial was conducted from July 2019 to December 2020, and cessation was due to the completion of subject recruitment. A total of 578 potentially eligible subjects were originally included. In total, 28 participants withdrew from the study or canceled their colonoscopy and eight received incomplete colonoscopies (Figure 1). Therefore, all outcomes were compared by ITT analysis in 550 subjects (275 for the formula LRD group vs. 275 for the self-prepared LRD group), and outcomes related to colonoscopy were compared by PP analysis in 542 subjects (273 vs. 269). There were no statistically significant differences in terms of baseline demographic and clinical characteristics between the two groups (Table 1).

**Table 1:** Demographic and clinical baseline characteristics of subjects.

**Characteristic**	**Formula LRD group (*n* = 275)**	**Self-prepared LRD group (*n* = 275)**	***P***
**Gender**			0.142
Male	168 (61.1)	151 (54.9)	
Female	107 (38.9)	124 (45.1)	
Age, years	47.24 ± 13.53	46.63 ± 12.57	0.581
Body mass index, kg/m^2^	23.21 ± 2.89	23.44 ± 3.09	0.376
**Indication**			0.900
Diagnostic colonoscopy	210 (76.4)	206 (74.9)	
Screening colonoscopy	51 (18.5)	53 (19.3)	
Surveillance colonoscopy	14 (5.1)	16 (5.8)	
**Anesthesia colonoscopy**			0.131
Yes	91 (33.1)	108 (39.3)	
No	184 (66.9)	167 (60.7)	
**Smoking** ^*^			0.097
Yes	62 (22.5)	79 (28.7)	
No	213 (77.5)	196 (71.3)	
**Alcohol consumption** ^#^			0.355
Yes	79 (28.7)	89 (32.4)	
No	196 (71.3)	186 (67.6)	
**Degree of education**			0.404
Poor (primary school or lower)	41 (14.9)	40 (14.5)	
Moderate (middle school)	120 (43.6)	135 (49.1)	
High (college or university)	114 (41.5)	100 (36.4)	

LRD, low-residue diet.

* Smoke more than one cigarette every day for more than 1 year.

# Drink alcohol of any type more than once every week for more than 1 year.

### Quality of bowel preparation

In ITT analysis, the formula LRD group showed a significantly higher adequate bowel preparation rate (94.5 vs. 80.4%; *P* < 0.001) and excellent bowel preparation rate (34.5 vs. 12.4%; *P* < 0.001), compared with the self-prepared LRD group. Significantly higher BBPS scores were observed in the formula LRD group in total score (7.87 ± 1.13 vs. 6.75 ± 1.47; *P* < 0.001), right colon (2.38 ± 0.61 vs. 2.00 ± 0.63; *P* < 0.001), transverse colon (2.75 ± 0.48 vs. 2.36 ± 0.63; *P* < 0.001), and left colon (2.74 ± 0.45 vs. 2.39 ± 0.57; *P* < 0.001). The distribution of total BBPS scores in the two groups was significantly different (*P* < 0.001). Results of the comparison of the quality of bowel preparation are shown in Table 2.

**Table 2:** Comparison of quality of bowel preparation by ITT analysis.

**Outcomes**	**Formula LRD group (*n* = 275)**	**Self-prepared LRD group (*n* = 275)**	***P***
Adequate bowel preparation rate	260 (94.5)	221 (80.4)	< 0.001
Excellent bowel preparation rate	95 (34.5)	34 (12.46)	< 0.001
**BBPS scores**			
Right colon	2.38 ± 0.61	2.00 ± 0.63	<0.001^*^
Transverse colon	2.75 ± 0.48	2.36 ± 0.63	<0.001^*^
Left colon	2.74 ± 0.45	2.39 ± 0.57	<0.001^*^
Total scores	7.87 ± 1.13	6.75 ± 1.47	<0.001^*^
**Distribution of BBPS scores**			<0.001
9	95 (34.5)	34 (12.4)	
8	93 (33.8)	56 (20.4)	
7	57 (20.7)	61 (22.2)	
6	20 (7.3)	83 (30.2)	
5	8 (2.9)	20 (7.3)	
4	0	15 (5.5)	
3	2 (0.01)	5 (1.8)	
2	0	1 (0.4)	
1	0	0	
0	0	0	

LRD, low-residue diet; BBPS, the Boston bowel preparation scale.

* The *p*-value was calculated by the Wilcoxon–Mann–Whitney test.

### Second outcomes

Other secondary outcomes of the ITT analysis are compared in Table 3. The dietary compliance rate was significantly higher in the formula LRD group than that of the self-prepared LRD group (92.4 vs. 78.9%; *P* < 0.001). It should be noted that 21 subjects in the formula LRD group did not adhere to the formula LRD, but nine of them were still considered to adhere to an LRD because they only consumed rice porridge or noodles instead on the day before colonoscopy.

**Table 3:** Comparison of secondary outcomes by ITT analysis.

**Outcomes**	**Formula LRD group (*n* = 275)**	**Self-prepared LRD group (*n* = 275)**	***P***
Dietary compliance rate	254 (92.4)	217 (78.9)	<0.001
**Tolerance**			
Degree of hunger	8.11 ± 2.15	5.98 ± 2.50	<0.001^*^
Intensity of physical strength	8.36 ± 1.69	6.36 ± 2.33	<0.001^*^
Influence on daily activities	8.33 ± 2.42	6.57 ± 2.33	<0.001^*^
Satisfaction	8.56 ± 1.61	7.20 ± 2.02	<0.001^*^
PEG compliance rate	268 (97.5)	258 (93.8)	0.037
**Adverse reactions**			
Nausea	32 (11.6)	39 (14.2)	0.373
Vomiting	17 (6.2)	21 (7.6)	0.501
Bloating	23 (8.4)	19 (6.9)	0.521
Abdominal pain	9 (3.3)	7 (2.5)	0.612
Insomnia	8 (2.9)	15 (5.5)	0.136
Allergy	0 (0)	1 (0.4)	>0.999
Cecal intubation rate	273 (99.3)	269 (97.8)	0.285
Cecal intubation time, minutes	6.17 ± 4.49	6.84 ± 5.74	0.124
Withdrawal time, minutes	8.07 ± 2.71	7.79 ± 3.29	0.282
Adenoma detection rate	70 (25.5)	44 (16.0)	0.006

LRD, low-residue diet.

* The *p*-value was calculated by the Wilcoxon–Mann–Whitney test.

The polyethylene glycol compliance rate was significantly higher in the formula LRD group compared with the self-prepared LRD group (97.5 vs. 93.8%; *P* = 0.037). In terms of tolerance, subjects in the formula LRD group showed a significantly lower degree of hunger (8.11 ± 2.15 vs. 5.98 ± 2.50; *P* < 0.001), a significantly higher intensity of physical strength (8.36 ± 1.69 vs. 6.36 ± 2.33; *P* < 0.001), and a significantly lower negative influence on daily activities (8.33 ± 2.42 vs. 6.57 ± 2.33; *P* < 0.001). The formula LRD group also showed significantly higher satisfaction (8.56 ± 1.61 vs. 7.20 ± 2.02; *P* < 0.01) with the whole bowel preparation process. There were no significant differences between the two groups in cecal intubation rate (99.3 vs. 97.8%; *P* = 0.285), cecal intubation time (6.17 ± 4.49 vs. 6.85 ± 5.74; *P* = 0.124), withdrawal time (8.07 ± 2.71 vs. 7.79 ± 3.29; *P* = 0.282), and adverse events. The ADR in the formula LRD group was significantly higher than that of the self-prepared LRD group (25.5 vs. 16.0%; *P* = 0.006).

### PP analysis

In PP analysis, the formula LRD group showed significantly higher adequate bowel preparation rate (95.2 vs. 82.2%; *P* < 0.001), excellent bowel preparation rate (34.8 vs. 12.6%; *P* < 0.001), BBPS scores in total score (7.93 ± 1.00 vs. 6.80 ± 1.41; *P* < 0.001), right colon (2.43 ± 0.52 vs. 2.02 ± 0.61; *P* < 0.001), transverse colon (2.77 ± 0.42 vs. 2.38 ± 0.61; *P* < 0.001), and left colon (2.73 ± 0.45 vs. 2.41 ± 0.56; *P* < 0.001). There were no significant differences between the two groups in cecal intubation time (6.18 ± 4.49 vs. 6.88 ± 5.78; *P* = 0.117), withdrawal time (8.11 ± 2.66 vs. 7.81 ± 3.29; *P* = 0.246), and in adverse events. The ADR in the formula LRD group was significantly higher than that of the self-prepared LRD group (25.6 vs. 16.0%; *P* = 0.006). The results of the PP analysis are shown in Table 4.

**Table 4:** Comparison of outcomes by PP analysis.

**Outcomes**	**Formula LRD group (*n* = 273)**	**Self-prepared LRD group (*n* = 269)**	***P***
Adequate bowel preparation rate	260 (95.2)	221 (82.2)	<0.001
Excellent bowel preparation rate	95 (34.8)	34 (12.6)	<0.001
**BBPS scores**			
Right colon	2.43 ± 0.52	2.02 ± 0.61	<0.001^*^
Transverse colon	2.77 ± 0.42	2.38 ± 0.61	<0.001^*^
Left colon	2.73 ± 0.45	2.41 ± 0.56	<0.001^*^
Total scores	7.93 ± 1.00	6.80 ± 1.41	<0.001^*^
Cecal intubation time, minutes	6.18 (4.49)	6.88 (5.78)	0.117
Withdrawal time, minutes	8.11 (2.66)	7.81 (3.29)	0.246
Adenoma detection rate	70 (25.6)	43 (16.0)	0.006

LRD, low-residue diet; BBPS, the Boston bowel preparation scale.

* The *p*-value was calculated by the Wilcoxon–Mann–Whitney test.

### Acceptability of the prepackaged formula LRD

Of the 275 participants who received the prepackaged formula LRD, 99 (36.0%) rated its taste as excellent, 118 (42.9%) as good, 55 (20.0%) as average, and only 3 (1.1%) as poor.

### Subgroup analysis

In subjects aged 45 years or older, the formula LRD group showed significant improvement in terms of adequate bowel preparation rate (93.3 vs. 81.5%; *P* = 0.002), excellent bowel preparation rate (29.7 vs. 14.1%; *P* = 0.001), BBPS scores (*P* < 0.01 in total scores, right colon, transverse, and left colon), dietary compliance rate (95.8 vs. 76.3%; *P* < 0.001), tolerance (*P* < 0.01 in degree of hunger, intensity of physical strength, and negative influence on daily activities), satisfaction (8.79 ± 1.39 vs. 6.97 ± 2.08; *P* < 0.001), PEG compliance rate (98.2 vs. 93.3%; *P* = 0.033), and ADR (34.5 vs. 21.5%; *P* = 0.014). There were no significant differences in cecal intubation rate (99.4 vs. 97.0%; *P* = 0.178), cecal intubation time (6.62 ± 4.59 vs. 6.30 ± 5.19; *P* = 0.571), and withdrawal time (8.70 ± 3.08 vs. 8.40 ± 3.64; *P* = 0.444) between the two groups. Results of the subgroup analysis of subjects aged 45 years or older are shown in Table 5.

**Table 5:** Subgroup analysis of subjects aged 45 years or older.

**Outcomes**	**Formula LRD group (*n* = 165)**	**Self-prepared LRD group (*n* = 135)**	***P***
Adequate bowel preparation rate	154 (93.3)	110 (81.5)	0.002
Excellent bowel preparation rate	49 (29.7)	19 (14.1)	0.001
**BBPS scores**			
Total scores	7.76 ± 1.09	6.74 ± 1.53	<0.001^*^
Right colon	2.32 ± 0.58	2.00 ± 0.67	<0.001^*^
Transverse	2.75 ± 0.44	2.36 ± 0.63	<0.001^*^
Left colon	2.70 ± 0.47	2.39 ± 0.56	<0.001^*^
Dietary compliance rate	158 (95.8)	103 (76.3)	<0.001
**Tolerance**			
Degree of hunger	8.26 ± 2.18	6.44 ± 2.01	<0.001^*^
Intension of physical strength	8.32 ± 2.50	6.75 ± 2.53	<0.001^*^
Influence on daily activities	8.50 ± 1.63	6.62 ± 2.05	<0.001^*^
Satisfaction	8.79 ± 1.39	6.97 ± 2.08	<0.001^*^
PEG compliance rate	162 (98.2)	126 (93.3)	0.033
Cecal intubation rate	164 (99.4)	131 (97.0)	0.178
Cecal intubation time, minutes	6.62 ± 4.59	6.30 ± 5.19	0.571
Withdrawal time, minutes	8.70 ± 3.08	8.40 ± 3.64	0.444
Adenoma detection rate	57 (34.5)	29 (21.5)	0.014

LRD, low-residue diet; BBPS, the Boston bowel preparation scale.

* The *p*-value was calculated by the Wilcoxon–Mann–Whitney test.

Subgroup analysis of ADR for different indications showed that the ADR of the formula LRD group was significantly higher than that of the self-prepared LRD group in diagnostic colonoscopy (23.1 vs. 14.4%; *P* = 0.025) and screening colonoscopy (35.3 vs. 17.3%; *P* = 0.038), while ADR between two groups in surveillance colonoscopy was not significantly different (28.6 vs. 31.3%; *P* = 1.000), as shown in Table 6.

**Table 6:** Subgroup analysis of adenoma detection rate for different indications.

**Indication**	**Formula LRD group**	**Self-prepared LRD group**	***P***
**Diagnostic colonoscopy**			
*N* = 409	208	201	
ADR, *n* (%)	48 (23.1)	29 (14.4)	0.025
**Screening colonoscopy**			
*N* = 103	51	52	
ADR, *n* (%)	18 (35.3)	9 (17.3)	0.038
**Surveillance colonoscopy**			
*N* = 30	14	16	
ADR, *n* (%)	4 (28.6)	5 (31.3)	>0.999

LRD, low-residue diet.

## Discussion

The fecal residue containing indigestible food material, microorganisms, secretions, and desquamated intestinal cells was discharged from the human gastrointestinal tract as feces (9). As fiber-containing foods produce the bulk of the fecal residue, reducing or even eliminating dietary fiber intake is crucial for bowel preparation (8). To achieve optimal bowel preparation results, the strict dietary restriction was often adopted. However, low calories and nutritional deficiencies in a strict dietary restriction may lead to poor compliance and tolerance symptoms, such as hunger, fatigue, dizziness, incomplete intake of laxatives, and even hypoglycemia and syncope, which may affect the quality of bowel preparation and even trigger patient resistance to next colonoscopy (14, 17). To change this situation, LRD was introduced as a method to balance nutritional intake with intake restriction of dietary fiber. Several previous studies have also demonstrated that LRD significantly improves patient tolerance without a negative affect on the quality of bowel preparation (14, 15, 17, 18).

A diet was commonly considered to be low residue when the total dietary fiber intake was <10 g per day (1, 9). European Society of Gastrointestinal Endoscopy (ESGE) recommends examples of foods allowed in a low-residue diet including some fresh peeled and pitted fruits and cooked vegetables (e.g., apples and carrots) (1). However, the aforementioned examples only cover some food types and there are many other foods meeting the criterion of <10 g of total dietary fiber intake per day. Therefore, patient compliance is often low when they prepare LRDs for themselves. A research reported that even after detailed dietary guidance by medical staff, only 44.2% of patients strictly adhered to an LRD (19). Prepackaged LRDs were designed to improve patient compliance and have been demonstrated to be effective (10–13), but the prepackaged LRDs used in these prior studies, composed of traditional foods, may need further improvement. The formula LRD adopted in the trial can be regarded as an improved prepackaged LRD. The powdered formula LRD contained no dietary fiber, which laid the foundation for achieving high-quality bowel preparation. In addition, sufficient energy and nutrients of the formula LRD ensured high tolerance. As for the self-prepared LRD group, the diet was restricted to a small range including rice porridge, rice soup, noodles, and eggs according to the dietary habits of the Chinese people to improve compliance. Eggs contain no dietary fiber, and 100 g of rice and 100 g of noodles contain an average of 0.7 and 0.8 g of dietary fiber, respectively. It was the low dietary fiber content of these foods that largely ensured that the total intake of dietary fiber of the self-prepared LRD was <10 g per day.

Polyethylene glycol plus simethicone was adopted as a catharsis regimen because PEG is currently the most widely used laxative and simethicone can reduce air bubbles in the intestine (20, 21). As for the PEG dose, the 3 L PEG split dose regimen was adopted as recommended by Chinese guidelines for bowel preparation for colonoscopy. ADR is an important quality indicator of colonoscopy and higher ADR is associated with lower incidence and mortality of CRC (22, 23). For safety, only adults younger than 65 years old were included in the study, and ADR was relatively low due to the relatively low average age of the two groups. However, in the subgroup of subjects aged 45 years or older, ADR increased obviously due to the increase in average age, from 25.5 to 34.5% in the formula LRD group and from 16.0 to 21.5% in the self-prepared LRD group. The age of 45 was chosen as a cut-off because that is a recommended initiating age for CRC screening (24–26). Through the subgroup analysis, we were able to preliminarily explore the effect of the prepackaged formula LRD on CRC screening in an age-appropriate population.

In addition, the subgroup analysis of ADR for different indications also showed higher ADR in diagnostic colonoscopy and screening colonoscopy. While in surveillance colonoscopy, the sample size (14 in the formula LRD group and 16 in the self-prepared LRD group) was too small to be representative, and the statistical result was not meaningful. Therefore, according to the results of subgroup analysis, the formula LRD played a significant role in diagnostic colonoscopy and screening colonoscopy, but its role in surveillance colonoscopy still needs to be further validated with a larger sample size.

Taste is an important indicator of diet acceptability. The questionnaire survey showed that nearly 80% of participants in the formula LRD groups evaluated the taste of the formula LRD as good or excellent, and only ~1% as poor, which indicated that the acceptability of the formula diet was fairly good.

Cost is an important factor affecting the acceptance of prepackaged formula LRD. Although the prepackaged formula LRD showed higher adequate bowel preparation rate and colonoscopy quality, its additional cost should not be ignored. At present, its cost is about US $40. The main raw material of the LRD is cheap soybeans, and its overall cost is expected to gradually decrease with the gradual reduction in processing cost.

There were still several limitations in the study. First, only one bowel preparation scale was used in this study, although it has been widely used, while using multiple scale scores may be more objective. Second, as a multicenter study, although unified training was conducted, there might be inevitable bias in BBPS scoring between researchers from various centers. Finally, the exclusion criteria excluded factors that might affect the quality of bowel preparation in order to straightly compare the effect of interventions on bowel preparation quality. The effect of the formula LRD on patients with these factors such as constipation merits further research.

## Conclusion

In summary, compared with self-prepared LRD, the prepacked formula LRD showed higher bowel preparation quality, compliance, and tolerance in bowel preparation. No new safety concern was observed in the prepacked formula LRD. More formula LRDs could be designed according to different dietary habits and ethnic populations, and further researches are warranted to confirm their effect.

## Data availability statement

The raw data supporting the conclusions of this article will be made available by the authors, without undue reservation.

## Ethics statement

The studies involving human participants were reviewed and approved by Ethics Committee of Shanghai Changhai Hospital. The patients/participants provided their written informed consent to participate in this study.

## Author contributions

ZL, YB, and PP contributed to the conception or design of the study. YB, PP, LG, SW, JM, HF, YC, SH, ZT, LX, ZF, YL, ZY, LY, WW, QH, TL, CL, DT, XW, YG, and HS contributed to the acquisition data of the study. YB, PP, and SZ contributed to the analysis and interpretation of the data of the study. YB and PP contributed to drafting the study or revising it critically for important intellectual content. ZL and YB agreed to be accountable for all aspects of the study in ensuring that questions related to the accuracy or integrity of any part of the study are appropriately investigated and resolved. All authors contributed to the article and approved the submitted version.
