# Supplementary material for: Prepackaged formula low-residue diet vs. self-prepared low-residue diet before colonoscopy: A multicenter randomized controlled trial
Source: Front Med (Lausanne). 2023 Mar 24;10:1029493. doi: 10.3389/fmed.2023.1029493 (PMC10079983; doi:10.3389/fmed.2023.1029493)
Supplement: Supplementary file 1 [file Table_1.DOCX]

**Nutrition Information of the prepackaged formula low-residue diet.**

| Nutrients | Unit | Per 100g | Per 100kj | Per pack |
| --- | --- | --- | --- | --- |
| Energy | kj | 1817 | 100 | 1090 |
| Dietary fiber | g | 0 | 0 | 0 |
| Protein | g | 20.0 | 1.1 | 12 |
| Fat | g | 13.0 | 0.7 | 7.8 |
| Linoleic acid | g | 2.1 | 0.12 | 1.3 |
| Flax acid | mg | 300 | 16.5 | 180 |
| Carbohydrate | g | 58.6 | 3.2 | 35.2 |
| Vitamin A | ug RE | 356 | 19.6 | 214 |
| Vitamin D | ug | 4.7 | 0.26 | 2.8 |
| Vitamin E | mg α-TE | 11.4 | 0.63 | 6.84 |
| Vitamin K_1_ | ug | 26.0 | 1.43 | 15.6 |
| Vitamin B_1_ | mg | 1.40 | 0.08 | 0.84 |
| Vitamin B_2_ | mg | 1.30 | 0.07 | 0.78 |
| Vitamin B_6_ | mg | 0.90 | 0.05 | 0.54 |
| Vitamin B_12_ | ug | 4.40 | 0.24 | 2.64 |
| Nicotinic acid | mg | 15.10 | 0.83 | 9.06 |
| Folic acid | ug | 135.0 | 7.4 | 81.0 |
| Pantothenic acid | mg | 6.20 | 0.34 | 3.72 |
| Vitamin C | mg | 120.0 | 6.6 | 72.0 |
| Biotin | ug | 11.0 | 0.6 | 6.6 |
| Na | mg | 400 | 22 | 240 |
| K | mg | 560 | 31 | 336 |
| Cu | ug | 257 | 14 | 154 |
| Mg | mg | 150 | 8.3 | 90 |
| Fe | mg | 5.0 | 0.28 | 3.0 |
| Zn | mg | 4.5 | 0.2 | 2.7 |
| Mn | ug | 560 | 30.8 | 336 |
| Ca | mg | 300 | 17 | 180 |
| P | mg | 246 | 13.5 | 148 |
| I | ug | 45.0 | 2.5 | 27.0 |
| Cl | mg | 557 | 31 | 334 |
| Se | ug | 20.0 | 1,1 | 12.0 |
